# Comparing, Contrasting, and Integrating Dissemination and Implementation Outcomes Included in the RE-AIM and Implementation Outcomes Frameworks

**DOI:** 10.3389/fpubh.2020.00430

**Published:** 2020-09-02

**Authors:** Kathryn Louise Reilly, Sarah Kennedy, Gwenndolyn Porter, Paul Estabrooks

**Affiliations:** ^1^School of Medicine and Public Health, University of Newcastle, Callaghan, NSW, Australia; ^2^The Priority Research Centre for Physical Activity and Nutrition, School of Education, University of Newcastle, Callaghan, NSW, Australia; ^3^University of Nebraska Medical Center, Omaha, NE, United States

**Keywords:** translational reseach, Implementation Outcomes Framework, scale-up, implementation outcomes, RE-AIM (Reach, Effectiveness, Adoption, Implementation and Maintenance)

## Abstract

As the field of dissemination and implementation science matures, there are a myriad of outcomes, identified in numerous frameworks, that can be considered across individual, organizational, and population levels. This can lead to difficulty in summarizing literature, comparing across studies, and advancing translational science. This manuscript sought to (1) compare, contrast, and integrate the outcomes included in the RE-AIM and Implementation Outcomes Frameworks (IOF) and (2) expand RE-AIM indicators to include relevant IOF dissemination and implementation outcomes. Cross tabular comparisons were made between the constitutive definitions of each construct, across frameworks, to reconcile apparent discrepancies between approaches and to distinguish between implementation outcomes and implementation antecedents. A great deal of consistency was identified across approaches, including adoption (the intention, initial decision, or action to employ an evidence-based intervention), fidelity/implementation (the degree to which an intervention was delivered as intended), organizational maintenance/sustainability (extent to which a newly implemented treatment is maintained or institutionalized), and cost. The IOF construct of penetration was defined as a higher-order construct that may encompass the reach, adoption, and organizational maintenance outcomes within RE-AIM. Within the IOF approach acceptability, appropriateness, and feasibility did not match constitutive definitions of dissemination or implementation but rather reflected theoretical antecedents of implementation outcomes. Integration of the IOF approach across RE-AIM indicators was successfully achieved by expanding the operational definitions of RE-AIM to include antecedents to reach, adoption, implementation, and organizational maintenance. Additional combined metrics were also introduced including penetration, individual level utility, service provider utility, organizational utility, and systemic utility. The expanded RE-AIM indicators move beyond the current approaches described within both the RE-AIM framework and IOF and provides additional planning and evaluation targets that can contribute to the scientific field and increase the translation of evidence into practice.

## Introduction

As the field of dissemination and implementation science matures, there are a myriad of outcomes that can be considered across individual, organizational, and population levels (1–4). The RE-AIM (Reach, Effectiveness, Adoption, Implementation, Maintenance) Framework was one of the first outcomes-focused approaches to address individual and organizational factors that would, if assessed and optimized, improve the generalizability of efficacy trials, and speed the translation of evidence-based interventions into sustained practice (5, 6). More recently, Proctoret al. introduced the Implementation Outcomes Framework (IOF)—specific to dissemination and implementation trials (4). Across these two approaches, 12 dissemination and implementation outcomes are proposed—some are distinct, some overlap, and some are duplicated—which can lead to difficulty in summarizing literature, comparing across studies, and advancing translational science.

The RE-AIM Framework includes 6 dimensions that focus planning and evaluation on balancing internal and external validity to develop intervention approaches that can achieve a public health impact (see Table 1). The framework includes dissemination outcomes at the individual (i.e., patient; reach) and organizational (i.e., adoption) levels. It also includes implementation outcomes that are operationalized at the organizational level (i.e., implementation and organizational maintenance). Finally, clinical outcomes are operationalized at the patient/participant level (i.e., effectiveness, maintenance). The overarching planning and evaluation goals of RE-AIM could be described as developing and testing interventions that (1) have the potential to reach a large and representative proportion of the intended audience, (2) effectively improve and sustain positive health outcomes, (3) have high adoptability across a large and representative proportion of the population of staff and settings intended to enact the intervention, (4) can be consistently implemented with a high degree of fidelity to underlying evidence-based principles at a reasonable cost, and (5) can be sustained in typical clinical or community settings (8).

**Table 1:** Definitions of IOF and RE-AIM outcomes.

**Implementation Outcome**	**Definition**
a. Acceptability	IOF: The perception among implementation stakeholders that a given treatment, service, practice, or innovation is agreeable, palatable, or satisfactory RE-AIM: N/A
b. Adoption	IOF: The intention, initial decision, or action to try or employ an innovation or evidence-based practice RE-AIM: The number, proportion, and representativeness of organizations or settings that agree to deliver the intervention, as well as the number, proportion, representativeness, and expertise of individuals in those settings that would ultimately deliver the intervention
c. Appropriateness	IOF: The perceived fit, relevance, or compatibility of the innovation or evidence-based practice for a given practice setting, provider, or consumer; and/or perceived fit of the innovation to address a particular issue or problem RE-AIM: N/A
d. Attributable individual level impact	IOF: N/A RE-AIM: Population Prevalence X Individual Level Impact (see Row l. below for definition)
e. Attributable organizational level impact	IOF: N/A RE-AIM: Population Prevalence X Organizational Level Impact (see Row o. below for definition)
f. Composite individual and organizational level impact	IOF: N/A RE-AIM: Reach + Effectiveness (or individual level Maintenance) + Adoption + Implementation/4 Maintenance (see below for detailed equations)
g. Costs	IOF: The cost impact of an implementation effort and of implementation strategies RE-AIM: Costs related to implementation and cost-effectiveness assessment
h. Effectiveness	IOF: N/A RE-AIM: A measurement of the degree to which the intervention is producing its intended effects while assessing potential unintended consequences and changes in quality of life
i. Feasibility	IOF: The extent to which a new treatment, or an innovation, can be successfully used or carried out within a given agency or setting RE-AIM: N/A
j. Fidelity	IOF: The degree to which an intervention was implemented as it was prescribed in the original protocol or as it was intended by the program developers RE-AIM: A component of implementation
k. Implementation	IOF: Aligns with Fidelity RE-AIM: Measures of cost and the degree to which the intervention is implemented with fidelity
l. Individual level impact (RE1)	IOF: N/A RE-AIM: Reach X composite Effectiveness = (participation rate - median ES_differential characteristics_) X (median ES_key outcomes_ - median ES_negative outcomes_ - median ES_differential impact_)
m. Individual level impact efficiency	IOF: N/A RE-AIM: (Incremental cost of treatment - control)/(incremental RE1 of treatment - control)
n. Maintenance	IOF: Included as sustainability RE-AIM: Considered at both the individual (maintenance of health outcomes ≥6 months post-intervention) and the setting (the degree to which the intervention has been institutionalized or sustainably adopted) levels
o. Organizational level impact AI1	IOF: N/A RE-AIM: (Organizational adoption rate - median ES_differential setting characteristics_) X (staff adoption rate X median ES_differential staff characteristics_) X (median component implementation rate across staff and Tx components - median ES_differential implementation_)
p. Penetration	IOF: The integration of a practice within a service setting and its subsystems. Later definitions included integration within service recipients (i.e., reach) (7) RE-AIM: A component of adoption and, if service recipients included, reach
q. Reach	IOF: Included if service recipients included in penetration RE-AIM: The absolute number, proportion, and representativeness of individuals who are willing to participate in a given initiative, intervention, or program, and reasons why or why not
r. Sustainability	IOF: The extent to which a newly implemented treatment is maintained or institutionalized within a service setting's ongoing, stable operations RE-AIM: Included as organizational maintenance of intervention implementation or institutionalization

Conceptualization of the RE-AIM framework has evolved over the past 20 years (6) to include a focus on qualitative research (1), cost across dimensions (9), and use in hybrid effectiveness-implementation research (10, 11). RE-AIM also provides composite metrics that address different aspects of intervention impact (Table 1) (12). Specifically, individual level impact can be determined using a composite measure of reach and effectiveness/maintenance using participation rate weighted by representativeness and standardized effect size weighted by differential effects across population subgroups. Attributable individual level impact can be determined by including population prevalence in the equation. Efficiency of individual level impacts was also proposed using the cost per unit of reach by effectiveness. Setting level impact measures can be calculated combining adoption rates and implementation fidelity—again with an option to include cost differentials. Finally, an overall summary index can be calculated by including composite equations for reach, effectiveness (or individual level maintenance), adoption, and implementation (12).

The IOF presents eight implementation outcomes, including: acceptability, adoption, appropriateness, costs, feasibility, fidelity, penetration, and sustainability (Table 1) (4). The IOF outcomes were conceptualized to improve the quality of dissemination and implementation trials, through the inclusion of measurable outcomes to enhance understanding of implementation success and processes. The IOF was also developed to distinguish between implementation, service, and client outcomes, develop a taxonomy of implementation outcomes, and highlight relationships across implementation outcomes at various stages in implementation research. For example, when considering the phases proposed in the Exploration, Preparation, Implementation, and Sustainment Model (EPIS) (13) differential levels of salience are proposed for each outcome. Acceptability, appropriateness, feasibility, and cost were considered most salient during the exploration phase, though each were also considered to have a lower degree of salience during preparation (i.e., appropriateness, feasibility), implementation (i.e., acceptability, cost), and sustainment (i.e., acceptability, cost) (7). Adoption was the only outcome considered to be most salient during preparation and was not considered salient at any other phase of implementation research. The outcomes that were considered to have primary salience for the implementation phase included fidelity and penetration while fidelity and sustainability were considered the most salient factors for the sustainment phase (7).

Similar to the RE-AIM framework, the conceptualization of IOF outcomes has also evolved over the 9 years since its first publication (4, 7). Of note, the concept of feasibility at the organizational level was extended to include feasibility at the service recipient level (7). Similarly, penetration was described as conceptually similar to reach and some researchers have extended the definition to include service recipients in addition to the service setting and its subsystems (7). Finally, similar to the RE-AIM framework the definition of cost has been refined to include cost of implementation (4), incremental costs (7), and overall financial impact of implementation efforts (14).

Both the RE-AIM framework and the IOF have had a significant impact on the field of implementation and dissemination science. RE-AIM provides a systematic planning and evaluation model that is based on individual and organizational outcomes, while the IOF provides conceptual clarity to distinguish between implementation, service, and client outcomes. Yet, there is considerable overlap between the frameworks and, based on the initial goals of the frameworks, key distinctions. This manuscript sought to compare, contrast and integrate dissemination, and implementation science outcomes included in these frameworks and provide working definitions that could extend the current RE-AIM indicators and outcome measurement approach.

## Methods

### Operationalization of RE-AIM and IOF Outcomes

Cross tabular comparisons were made between the constitutive definitions of the IOF and RE-AIM framework variables (see Table 1). Framework definitions were sourced from both the source papers as well as updated conceptualizations and models to increase the likelihood that definitions included advancements since initial publication (4, 6, 15). Using a content validity approach, all co-authors independently coded IOF constructs across the RE-AIM dimensions as either; (i) consistent between frameworks, (C); (ii) potential combined metrics (CM) (i.e., IOF construct aggregated across RE-AIM dimensions), or (iii) predictors (P) (or antecedents) of dissemination or implementation (Table 2). The study team met monthly between July and December 2019 to discuss and agree on coding for content analyses, to compare individual coding and resolve any discrepancies between team members through consensus. Coding of the framework definitions was completed by a senior scientist, post-doctoral fellow, and two doctoral candidates all specializing in dissemination and implementation science.

**Table 2:** Cross-tabular comparison of RE-AIM and IOF outcomes.

**Implementation outcome framework**	**RE-AIM**					**Level of analysis**
	**Reach**	**Effectiveness**	**Adoption**	**Implementation**	**Maintenance**	
Acceptability			P	P	P	O
Adoption			C			O
Appropriateness			P	P	P	O
Costs				C		O
Feasibility			P	P	P	O
Fidelity		P		C		O
Penetration	CM		CM		CM	O
Sustainability					C	O
Level of analysis	I	I	O	O	I / O	

*C, Consistent between frameworks; CM, Potential combined metric; P, Predictor of implementation/dissemination; I, Individual; O, Organizational*.

For the purpose of this comparison, we defined “predictors” as constructs that act as precursors to implementation and dissemination of evidence-based interventions. For example, an intervention would need to be perceived as acceptable in order for it to be adopted. Characterization as a predictor was based on the degree to which the construct definition aligned with constitutive definitions of dissemination (i.e., an active approach to spreading evidence-based interventions to a target audience) and implementation (i.e., the process of delivering or enacting evidence-based interventions according to protocol or principles) (15). The level of analysis operationalized as individual (reflecting service recipients, patients, participants) or organizational (reflecting staff, settings, and organizations) was also identified. Gaps identified across the RE-AIM framework and the IOF were also considered and addressed through a proposed expansion of the operational definitions of RE-AIM indicators. Specifically, while cost and adaptation have both been discussed and examined in the context of both frameworks—methods to operationalize both have been limited (4, 7, 16–19). Framework operational definitions based on the cross tabular comparisons are shown in Table 2.

## Results

### Operationalisation of RE-AIM and Implementation Outcomes

A great deal of consistency was identified across approaches, including adoption (i.e., the intention, initial decision, or action to try or employ an evidence-based intervention), fidelity/implementation (i.e., the degree to which an intervention was delivered as intended), organizational maintenance/sustainability (i.e., extent to which a newly implemented treatment is maintained or institutionalized), and cost. However, cost was more explicitly defined in the IOF as cost of an implementation effort and of any strategies that targeted improvements in implementation whereas the RE-AIM conceptualization of cost focused on implementation and cost-effectiveness. The IOF construct of penetration was defined as a higher-order construct that may encompass the reach, adoption, and sustainability outcomes within RE-AIM. Within the IOF approach there were also a number of constructs that reflect theoretical antecedents of implementation outcomes including acceptability, appropriateness, and feasibility—rather than reflecting the constitutive definitions of dissemination or implementation. Table 2 outlines the cross tabulation of the constructs of the IOF and RE-AIM frameworks.

### Expanded Operationalization of RE-AIM Dimensions

Integration of the IOF constructs with the RE-AIM framework was successfully achieved by extending the operational definitions of each RE-AIM dimension using IOF outcomes and antecedents. In addition, adaptation and cost considerations by RE-AIM dimension—both highlighted, but not explicitly included across dissemination and implementation outcomes were included in the expanded indicators; see Table 3. The dimensions of effectiveness and individual-level maintenance were the only RE-AIM components that were not expanded through this process. In addition, the IOF concepts of adoption and sustainability were identified as duplicates with the RE-AIM domains of adoption (staff/service provider- and organizational-level) and organizational-level maintenance, respectively, and were operationalized as such.

**Table 3:** Expanded operationalization of RE-AIM.

**Dimension**	**Expanded operational definition**
Reach	• Number of participants or individuals that participate in or are exposed to a clinical or public health intervention • Proportion of the intended audience that participate in or are exposed to a clinical or public health intervention • The representativeness of participants relative to the intended population that participate in or are exposed to a clinical or public health intervention • *Antecedent assessments—service recipient perceptions of:* ◦ *Appropriateness (IOF definition—consumer level)* ◦ *Acceptability (The perception among service recipients that a given treatment, service, practice, or innovation is agreeable, palatable, or satisfactory)* ◦ *Feasibility (The extent to which a new treatment, or an innovation, can be successfully used or carried out by a service recipient)* • Cost of dissemination strategies intended to increase participation of those whose health would benefit from the intervention^*^
Effectiveness	• The degree to which the intervention is producing its intended effects while assessing potential unintended consequences and changes in quality of life • Cost-benefit based on total intervention costs by magnitude of effectiveness • *No expansion proposed for this dimension*
Adoption	• Number of settings that participate in or are exposed to the public health intervention • Proportion of the intended settings and staff that deliver or are exposed to the public health intervention • Representativeness of settings relative to the intended population that participate in or are exposed to the public health intervention • *Antecedent assessments—organizational staff and stakeholder perceptions of:* ◦ *Acceptability (organizational satisfaction with various aspects of the public health intervention and intervention congruence with organizational mission)* ◦ *Appropriateness (IOF definition—organization or setting level)* ◦ *Feasibility (IOF definition– The extent to which a new treatment, or an innovation, can be successfully used or carried out within a given agency or setting)* • *Start-up cost assessment* • *Costs of dissemination strategies intended to increase participation of staff and settings in implementation of the EBI*
Implementation	• Consistency of delivery as intended and in the time required across staff and organizations • *Adaptation* ◦ *Assessing indicators of adaptation prior to, during, and following implementation of the intervention* ◦ *Document who, what, when, where, and why adaptations were made (18, 20)* ◦ *Document how the adaptation was consistent with the underlying evidence-based principles of the intervention as previously tested (20)* • *Antecedent assessments:* ◦ *Organizational experience of acceptability (organizational satisfaction with various aspects of the public health intervention and intervention congruence with organizational mission)* ◦ *Organizational experience of appropriateness (IOF definition—organization or setting level)* ◦ *Organizational experience of feasibility (IOF definition– The extent to which a new treatment, or an innovation, can be successfully used or carried out within a given agency or setting)* • *Cost of implementation* • *Cost of strategies targeting quality of implementation* • *Budget impact assessment*
Maintenance—individual level	• The extent to which the intervention's primary outcome is sustained ≥6 months after intervention completion • *No expansion recommended for this dimension*
Maintenance—organizational level	The public health intervention becomes institutionalized or part of the routine organizational practices and policies • *Antecedent assessments* ◦ *Experienced acceptability (Organizational satisfaction with various aspects of the public health intervention and intervention congruence with organizational mission)* ◦ *Experienced appropriateness (IOF definition—organization or setting level)* ◦ *Experienced feasibility of EBI to the intended staff and setting intended to implement*. • *Cost of sustained implementation* • *Cost of strategies targeting sustained implementation*
Combined metrics	• Individual-level impact: reach X effectiveness • Individual-level impact efficiency: incremental cost increases by unit of reach X effectiveness • Organizational level impact: adoption X implementation (or organizational maintenance) • Attributable individual-level impact: population prevalence X individual level impact • Attributable organizational-level impact: population prevalence X organizational level impact • Comprehensive individual/organizational impact: reach + effectiveness (or individual level maintenance) + adoption + implementation/4 maintenance • *Penetration: reach X adoption X organizational maintenance* • *Individual level utility: participant ratings of acceptability X appropriateness X feasibility* • *Service provider utility: implementation staff ratings of acceptability X appropriateness X feasibility* • *Organizational utility: organizational decision maker ratings of acceptability X appropriateness X feasibility* • *Systemic Utility: individual utility + service provider utility + organizational utility*

* *Text in Italics represents new components of each RE-AIM dimension*.

The IOF constructs of acceptability, appropriateness, and feasibility were included as multi-leveled variables across reach, adoption, implementation, and organizational maintenance. However, the level of application is hypothesized to differ by dimension and temporality of assessment of the construct relative to the initial implementation (e.g., before, during, and after). Specifically, initial perceptions of intervention acceptability, appropriateness, and feasibility were operationalized as unique antecedents of reach (i.e., individual-level; participants/patients) and adoption (i.e., staff, setting, organization-level; service providers/organizational decision makers). In each of these cases the temporal assessment of these constructs and the potential for predictive validity is hypothesized to be dependent on the initial perceptions of the intervention prior to individual (reach) or organizational (adoption) decisions on engagement or uptake. In contrast, organizational experience—indicating a later temporal assessment following the initial actions of implementation—of acceptability, appropriateness, and feasibility were hypothesized to be antecedents of implementation fidelity (i.e., staff, setting, organizational-level) and organizational-level maintenance.

Cost specification was also expanded across reach, adoption, implementation, and organizational maintenance outcomes. An overarching consideration included that for most outcomes at least two categories of costs could be assessed—the cost of a dissemination or implementation strategy used to enhance a specific RE-AIM dimension and the cost of completing the activities associated with each dimension. For example, an implementation strategy could include the cost of training staff on the intervention delivery and the cost of implementing the intervention itself. The training costs are distinct from the ongoing operational costs for intervention implementation. In addition to these two costs, specific budget impact assessments (16) are included to provide practical information for implementation sites.

Of note, adaptation was not included in the original operational definitions provided by the IOF and RE-AIM framework. Recently, however, there have been suggestions to advance the consideration of adaptation within the context of implementation (17). To address this need we expanded the implementation dimension to include assessing indicators of adaptation prior to, during, and following implementation of the intervention. Initial indicators were based on suggestions from Stirman-Wiltsey et al. to document who, what, when, where, and why adaptations were made (18, 20). In addition, we included documentation on how the adaptation was consistent with the underlying evidence-based principles of the intervention as previously tested (20).

The final area of expansion of RE-AIM indicators was in the realm of combined metrics. Penetration was operationalized to include the product of reach, adoption, and organizational maintenance to provide an overarching system-based outcome. Other expanded combined metrics focused on determining the utility of an intervention at the participant, service provider, and organizational decision-maker level. In each case, utility was defined as the product of ratings of acceptability, appropriateness, and feasibility. Each of these metrics were further combined as an aggregate rating to produce a measure of systemic utility.

## Discussion

This manuscript described the process used to compare, contrast and integrate dissemination and implementation science outcomes included in the RE-AIM framework and the IOF. We used a cross-tabular content analysis to compare between the frameworks which highlighted similarities and key differences. In addition, we integrated IOF within the context of the RE-AIM dimensions which generated an increased depth for a number of constructs and provided additional guidance on the possibility to examine combined metrics-particularly during later stages of scale-up activities. Based on this work we hypothesize that assessment of the expanded RE-AIM outcomes will improve the ability of dissemination and implementation scientists to document key outcomes that reflect the achievement of translating evidence-based interventions that promote public health.

The primary distinction between the two frameworks was an inclusion of individual level factors (RE-AIM) and predictors or antecedents of dissemination and implementation outcomes (IOF). The distinctions between these two models is not surprising when considering the rationale for the development of each (4, 5). The IOF was developed to better clarify dissemination and implementation outcomes for the specific field of dissemination and implementation research (4). In contrast, the RE-AIM framework was developed to be used across the translational spectrum of research and encourage some assessment of external validity in efficacy trials while also encouraging some assessment of internal validity in dissemination and implementation trials (21). The comparison between the frameworks allowed the consideration of variables that can be assessed at the individual, service-recipient level and those that can be assessed at an organizational and service provider level.

As Table 2 demonstrated, the primary overlap between the frameworks was within the organizational components of the RE-AIM framework. This highlighted a limitation of the IOF in the area of understanding a key dissemination outcome—reach. Reach, which can be considered an operationalization of consumer-demand for an evidence-based intervention, has been proposed as a key factor in organizational uptake and sustainability (22). While the explicit focus on reach may have been a limitation of the IOF, the focus on acceptability, appropriateness, and feasibility—albeit at the level of the service provider, organization, and organizational sub-systems—was a strength. We proposed that acceptability, appropriateness, and feasibility could be considered at multiple levels and at different temporal points across the translation research spectrum. First, these constructs would enhance the understanding of acceptability, appropriateness, and feasibility of service recipients. When applied to service recipients, the population that would have health benefits from the evidence-based intervention, understanding these variables can provide valuable information relative to the potential for an evidence-based approach to achieve high reach (23). By integrating these ideas within an expanded operationalization of RE-AIM indicators, it also provides additional planning and evaluation metrics that can heighten the likelihood of achieving broach reach when an intervention is taken to scale. Second, operationalizing these constructs temporally would entail the use of future and present tense language that could easily be applied to existing validated tools. For example, Weiner et al. (24) measures of intervention appropriateness and feasibility include temporal language appropriate for reach and adoption (e.g., this intervention seems doable) that could be adapted for prediction of ongoing implementation and organizational maintenance (e.g., this intervention is/was doable) reflecting experience in participation and delivery.

The newly expanded RE-AIM indicators has the potential to perform well due to its expanded definitions, in regards to assessment within staged research models such as the Pathways to Scale-Up Model (Pathways) (25), used primarily in Australia, to determine intervention readiness for broad application. “Pathways” describes four stages of scaling up evidence-based interventions: development, efficacy, effectiveness, and dissemination (25). As the RE-AIM framework was developed to be applicable across the translational research spectrum (21, 26), it has greater utility than the IOF for investigators using models such as “Pathways” —which can be applied to both evidence-based interventions as well as novel intervention approaches based on sound theory—and requires the assessment of service recipient outcomes (25). Similarly, the expanded RE-AIM metrics also may be ideal for hybrid effectiveness-implementation trials (27) that necessitate assessing effectiveness at the service recipient level and implementation at the service provider or organizational level. Contextual assessment is also a key component for hybrid type 1 trials that have a primary outcome of effectiveness. The assessment of context can include examining barriers and facilitators to future implementation efforts, potential for adoption and sustainability, and likelihood of high reach. The expanded RE-AIM metrics provide further contextual information related to acceptability, appropriateness, and feasibility that could advance understanding of how best to design implementation fit for the intended audience or service provider.

The assessment of cost was increased to move beyond cost of implementation and implementation strategies, cost effectiveness, and budget impact analysis to a more comprehensive assessment across RE-AIM dimensions. This aligns with the importance of a wide range of cost considerations used by policy makers and organizational leaders (16). The area of cost assessment and analysis in dissemination and implementation science is emerging (16, 19) and the expanded cost metrics provide a methodology for assessing costs related to reach, adoption, implementation, and organizational maintenance—with a focus on both the strategies used to enhance each outcome and the operational costs associated with each dimension. This will allow for the development of cost simulation models (28) that could vary dissemination and implementation strategy use and provide variable budget impact scenarios for systems considering the uptake of a new evidence-based intervention. For example, a new evidence-based diabetes prevention intervention, being introduced for community YMCAs could use dissemination and implementation strategies that include marketing strategies to increase adoption, participant incentives to increase reach, auditing and feedback processes to improve implementation, and a budget matrixing activity to improve likelihood of organizational maintenance. With the appropriate data on responsiveness of each RE-AIM outcome to the respective dissemination and implementation strategy, would allow a determination of the cost and impact with and without each strategy.

Adaptation was not explicitly defined in either the RE-AIM Framework or IOF, but is necessary to consider during the implementation of an intervention (6). Adaptations (i.e., changes to the intervention components, and delivery method) may elicit changes in the effectiveness of interventions (both positive and negative), and as such it is vital that these are noted and assessed when possible, serving as a useful insight into intervention components across the stages. Despite the potential for adaptations to alter effectiveness of interventions, there are several benefits that arise—such as addressing barriers to program adoption, implementation, and sustainment at the individual, service and organizational level (29). As noted by several authors, the key to determining the impact of adaptations is careful tracking and reporting of how, why, and by whom the adaptations were made and the resulting changes in individual and organizational outcomes (20, 30). It is of note that recent conceptual descriptions of adaptations related to the RE-AIM framework (31) highlighted the likelihood that adaptations are iterative and may be addressed across adoption, implementation, and sustainability—additional research in this area will help to determine at which points meaningful adaptation occurs.

The combined metrics proposed for the RE-AIM framework have not been broadly used across the extant public health or dissemination and implementation science literature. It is unclear whether this uptake is based on the lack of applicability of these metrics and/or the difficulty in gathering all the necessary data. Still, the original combined metrics provided an opportunity to consider a single number to assess individual and organizational impact (12). We proposed these metrics to allow for the scientific comparison of differential impact of various dissemination and implementation science strategies. Broader evaluation efforts that include attributable individual-level impact, penetration, and individual-level utility may help researchers and public health professionals better understand intervention reach and, if needed, adapt recruitment and retention efforts to improve individual-level engagement and sustained participation. This is also applicable to adoption at the service provider and system level. These combined metrics may provide additional, and potentially more practical, ways to assess utility at multiple levels and across time with relatively simple measures that can be proactively collected (24). Further, using the expanded RE-AIM outcomes may not only speed up the translation of evidence into practice, in an attempt to alleviate the stark difference that exists between research and policy timelines (32), but may also help researchers and policy makers to determine cost-impacts of interventions. For an intervention to be novel to policy makers, it needs to provide favorable outcomes at the individual and organizational level, aligned with their specific policy goals, as well as having cost benefit (33). The expanded RE-AIM indicators presented here moves beyond current approaches and provided additional planning and evaluations targets that can contribute to dissemination and implementation science and increase the translation of evidence into practice.

A potential limitation of our expanded RE-AIM approach is that, by including antecedents to dissemination and implementation outcomes we are initiating a shift from an outcome framework to a blended outcome framework and explanatory model (34). As such, the expanded outcomes we propose limit other factors that could provide explanation for specific reach, adoption, implementation, and organizational maintenance outcomes. For example, the Practical, Robust Implementation, and Sustainability Model (PRISM) evaluates the impact of a public health intervention on various domains of RE-AIM as they translate to real-world practice (35). The model considers organizational and patient perspectives of the intervention characteristics, drawing similarities to intervention beneficiary and organizational evaluations of acceptability, appropriateness, and feasibility—though they do not explicitly list these as potential constructs (35, 36). The expanded RE-AIM indicators presented here may simply set the stage to consider theoretically-compelling constructs that could be dissemination and implementation strategy targets to improve RE-AIM outcomes through theoretically derived mediators (37). Additionally, conducting concurrent validity testing on data collected on the indicators that are consistent between the RE-AIM and IOF frameworks would be valuable in a future study.

The expansion to RE-AIM indictors is intended to improve the planning and outcomes related to health-enhancing interventions. However, a potential unintended consequence of this paper is that it is counter to the intuitive nature of the RE-AIM framework (38). That is, by adding complexity to the breadth of RE-AIM indicators it could be a barrier to applying the framework. This paper highlights the similarities between RE-AIM and the IOF, pushes the boundaries of how best to consider dissemination and implementation outcomes, and provides opportunities for confirmation or rejection of the expanded RE-AIM indicators. It is hypothesized that the use of the expanded RE-AIM indicators across the dissemination and implementation research continuum may assist in speeding up the translation of evidence into practice—and advance the science surrounding that translation. Each of the proposed expansions should be examined, from a scientific and pragmatic perspective, to determine the salience of the indicators and metrics across research and practice stakeholder groups. Understanding the practicality, reliability, and validity of our approach will help to advance the planning and evaluation of future translational research studies focused on developing and testing evidence-based interventions.

## Data Availability Statement

The raw data supporting the conclusions of this article will be made available by the authors, without undue reservation.

## Author Contributions

KR, SK, GP, and PE equally contributed to the conceptualization of this report, analyzing definitions, compiling cross-tabular comparisons, and manuscript writing. All authors contributed to the article and approved the submitted version.

## Conflict of Interest

The authors declare that the research was conducted in the absence of any commercial or financial relationships that could be construed as a potential conflict of interest.
